# Karyotyping of circulating tumor cells for predicting chemotherapeutic sensitivity and efficacy in patients with esophageal cancer

**DOI:** 10.1186/s12885-019-5850-7

**Published:** 2019-07-03

**Authors:** Yingjie Chen, Zhipeng Yang, Yingxue Wang, Juandong Wang, Chuanxin Wang

**Affiliations:** 1 Department of Clinical Laboratory, The Second Hospital of Shandong University, Jinan, Shandong China; 2 Department of Hematology, The Second Hospital of Shandong University, Jinan, Shandong China

**Keywords:** Circulating tumor cells (CTCs), Esophageal cancer, Chemotherapeutic efficacy, Triploid/non-triploid patient

## Abstract

**Background:**

Aneuploidy of chromosome 8 in circulating tumor cells (CTCs) has been reported correlates with therapeutic efficacy and prognosis in patients with advanced gastric cancer. However, it is not clear whether it is also appropriate for other cancer. Therefore, in this study, we evaluate the clinical application aneuploidy of CTCs for esophageal cancer.

**Methods:**

Peripheral blood were collected for karyotyping analysis before and after first 4-cycles chemotherapy from seventy nine patients with newly diagnosed esophageal cancer. Karyotyping of chromosome 8 in CTCs detected by SET-iFISH (Subtraction Enrichment-Immunostaining fluorescence in situ hybridizatio) in those patients were grouped into two categories according to CTC number: triploid group and non-triploid group. Pearson Chi-Square were used to compare the association between different aneuploidy type and chemotherapeutic sensitivity and efficacy.

**Results:**

Among the 16 patients with triploid of chromosome 8, 4 patients benefit, and of the 63 patients with non-triploid, 54 patients benefit. Chi-square test analysis found that clinical benefit of non-triploid patients was significantly higher than triploid patients, suggesting non-triploid patients were more sensitive to chemotherapy than triploid patients. After 4-cycles chemotherapy, it is found that chemotherapeutic efficacy was positively correlated with non-triploid proportion. These results suggest that non-triploid proportion could be used as a candidate maker for assessing chemotherapeutic efficacy.

**Conclusions:**

Monitoring aneuploidy of chromosome 8 in CTCs before and after chemotherapy may help predict sensitivity and efficacy of chemotherapy in patients with esophageal cancer.

**Electronic supplementary material:**

The online version of this article (10.1186/s12885-019-5850-7) contains supplementary material, which is available to authorized users.

## Background

Esophageal cancer is a malignant tumor of the esophagus that presents as dysphagia. Is a serious life-threatening disease. Esophageal cancer is the eighth most common malignant tumor worldwide [1] and is the sixth leading cause of cancer death [2]. However, the early diagnosis of EC is difficult due to the lack of specific symptoms in the early stages. In most cases, the disease is already at an advanced stage at presentation. Surgical resection is the main treatment for EC, with a postoperative 5-year survival rate of only 34–36% [2]. Identification an early biomarker of chemotherapy efficacy for improving clinical treatment outcome of esophageal cancer patients is therefore of particular significance.

CTCs are tumor cells in the blood which comes from primary or metastatic solid tumors. The recurrence and metastasis supposedly result from clinically occult, minimal residual disease caused by circulating tumor cells (CTCs), or disseminated tumor cells [3].CTCs measurement as a non-invasive detection method which can monitor therapeutic responses of cancer patients dynamically [4–6]. Quantification of CTC not only can serve as a biomarker for cancer diagnosis but also they could be used for indicating cancer prognosis [7–11]. It has been reported that advanced gastric cancer patients in Japan with unfavorable CTC counts several weeks after initiation of chemotherapy had shorter median progression-free survival and overall survival compared with those with favorable CTC counts [12]. In follow-up of patients with advanced gastric cancer, quantification of CTCs may be used as a promising approach for evaluating chemotherapeutic efficacy and predicting prognosis of these patients [13].

In addition to counting CTCs, CTC subtype classified by specific tumor biomarker expression and/or chromosome ploidy could also be used as an alternative approach [14]. The phenotype and karyotyping analysis of CTC subtypes in patients with gastric cancer showed that CTCs with cytokeratins 18-negative/triploidy of chromosome 8 show endogenous resistance to cis-platinum, and CTCs with cytokeratins 18-positive/tetraploidy or over tetraploidy of chromosome 8 show endogenous acquired resistance to cis-platinum [15]. After chemotherapy, triploid CTCs showed intrinsic resistance to chemotherapeutic reagents, whereas multiploid CTCs (≥4 copies on chromosome 8) developing acquired resistance [16]. Therefore, identification of different CTCs subtypes associated with distinct clinical significance could help guide individual precision therapy.

In recent years, studies on peripheral blood CTCs have shown that CTCs can be used to predict the progress and prognosis of esophageal cancer, become an important biomarker for diagnosis, metastasis and recurrence, and an effective indicator to judge poor prognosis of patients. However, most of these studies were based on CTC count and phenotype detection. Recently, it has been reported that the increased copy number of chromosome 8 is significantly correlated with esophageal cancer and lymph node metastasis [17], but the relationship between CTC karyotype and therapeutic effect and prognosis of esophageal cancer has not been further elaborated.

In this study, we investigated whether and how distinct CTC subtypes with different ploidy of chromosome 8 in CTCs of esophageal cancer patients are correlated with chemotherapeutic efficacy. SET-iFISH measurement was applied to enrich and characterize ploidy of chromosome 8 in CTCs. The association between multiploid CTCs in patients before and after themotherapy and chemotherapeutic efficacy were analysed to monitor therapeutic response of esophageal cancer patients.

## Methods

### Patients and sample collection

Seventy nine patients with newly diagnosed esophageal cancer were enrolled in The Second Hospital of Shandong University from February 2015 to February 2018. Patients who had not been treated with chemotherapy were recruited in this study. Six ml peripheral blood were taken 2 weeks before chemotherapy. After then these patients received first-line PTX or DDP chemotherapy with 4 cycles, another 6 ml peripheral blood were taken 2 weeks after first 4-cycles chemotherapy.

The therapeutic effect of esophageal cancer patients was assessed after every 2 cycles of chemotherapy by computed tomography based on the RECIST1.1 criteria, including PR (Partial Response), PD (Progressive Disease), SD (Stable Disease).

This study was approved by the Human Research Ethical Committee of the Department of Clinical Laboratory, The Second Hospital of Shandong University, Jinan, Shandong, with protocol No.KYLL-2019 (LW)-001. Written informed consent was obtained from each participants included in this study.

### Detection of CTCs karyotyping by SET-iFISH

CTCs karyotyping was detected by SET-iFISH according to the manufacture’s updated instruction (Cytelligen, San Diego, CA, USA), as previously described [15].

### Subtraction enrichment

Briefly, ACD blood vessels were used to collect 6 ml of peripheral blood. All operations were performed at room temperature. Samples were kept and must be processed within 48 h. Samples were centrifuged at 200×g for 15 min. The supernatant was discarded. Then added buffer to make up 6 ml, followed by loading on the non-hematopoietic cell separation matrix in a 50 ml tube, and subsequent centrifugation at 450×g for 5 min. The supernatant and white membrane layer were collected into a 50 ml centrifuge tube containing white blood cells and tumor cells, and then incubated with 300 μl of immuno-magnetic beads conjugated to a cocktail of anti-leukocyte mAbs, for 30 min with gentle shaking. White blood cells were removed using a magnetic rack. The solution was added to a 15 ml centrifuge tube and 14 ml was replenished with buffer. The solution was centrifuged at 500×g for 5 min. Supernatants were aspirated down to 50 μl. Sedimented cells in 50 μl solution were gently resuspended, followed by subjection to immunofluorescence staining, and drying monolayer of cells mixed with the special fixative on the Cytelligen coated and formatted CTC slides for subsequent iFISH analyses.

### iFISH

Briefly, dried monolayer cells on the coated and formatted CTC slides were rinsed and incubated with PBS for 3 min, followed by hybridization with Vysis chromosome 8 centromere probe (CEP8) SpectrumOrange for 4 h. Samples were subsequently incubated with Alexa Fluor (AF) 488(green), 594(red), respectively conjugated to the mAbs recognizing indicated targets, including CD31, CD45, for 20 min in dark. After washing, samples were mounted with mounting media containing DAPI, and subjected to automated CTC image scanning and analyses.

CTCs identified by SET-iFISH are DAPI+ (blue)/FISH+ (aneuploid chromosome 8, range)/CD31- (green) and CD45-(red).

We define the karyotype based on the number of chromosomes 8.The number of chromosome 8 is 3, and the cell is triploidy; The number of chromosome 8 is 4, and the cell is tetraploidy; the number of chromosome 8 is 5, and the cell is pentaploidy; the number of chromosome 8 is more than 5, and the cell is multiploidy.

### Statistical analysis

SPSS 20.0 software was used to perform statistical analyses. The differences in karyotyping of CTC between triploid genotype patients and non-triploid genotype patients patients were compared by Chi-square test. Correlation between the karyotyping of CTC and chemotherapeutic efficacy were assessed by Spearman’s correlation. *P* < 0.05 represent statistically significant.

## Results

**Fig. 1:**
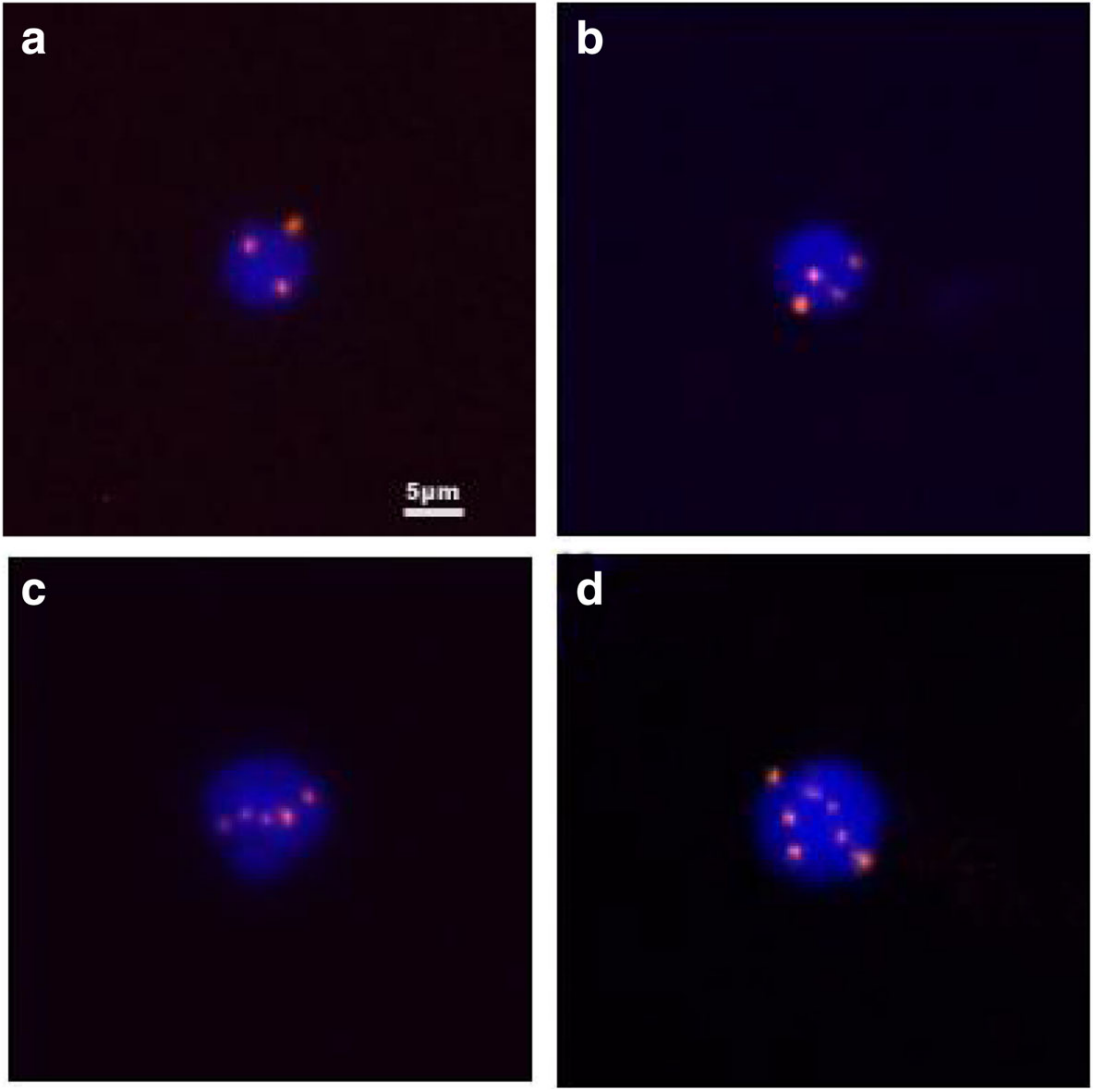
Images of CTCs with different ploidies of chromosome 8. (**a**) triploidy, (**b**) tetraploidy, (**c**) pentaploidy, and (**d**) > 5 copies. Nuclei of CTCs were stained with 4′, 6-diamidino-2-phenylindole (blue) and the chromosome 8 was identified by centromere probe 8 Spectrum Orange probe (red dots). Scale bar = 5 μm

As showed in Fig. 1, triploidy, tetraploidy, pentaploidy or greater than pentaploidy copies of chromosome 8 in CTCs were observed in esophageal cancer patients, indicating heterogeneous polysomic of chromosome 8 exist in CTCs. Esophageal cancer patients were classified according to karyotyping of chromosome 8. Patients with number of triploid CTC over or equal to 60% were classified as triploid genotype patients, while patients with less than 60% were classified as non-triploid genotype patients. According to above classified standard, of these 79 patients, 16 were triploid genotype patients, and 63 were non-triploid genotype patients.

The chemotherapeutic efficacy of the 79 patients were evaluated according to the RECIST1.1 standard after first 2-cycles of chemotherapy (Table 1 and Additional file 1: Table S1). The results showed that of these 16 triploid genotype patients, 10 were no-benefit group (PD), 6 were benefit group (PR and SD), and of these 63 non-triploid genotype patients, 9 were no-benefit group, 54 were benefit group (Table 2). Chi-square test analysis of the chemotherapeutic efficacy and patients’ ploidy showed that chemotherapeutic efficacy of non-triploid patients were significantly higher than triploid patients (*p* < 0.05), suggesting that the chemotherapy sensitivity of non-triploid patients were higher than triploid patients.

**Table 1:** CTC karyotyping and chemotherapeutic efficacy of 79 esophageal cancer patients with first 2-cycles chemotherapy

		Patient type	
		triploid	non-triploid
Chemotherapeutic efficacy	PR	0	31
	SD	4	23
	PD	12	9

Note: PR: Partial Response; PD: Progressive Disease; SD: Stable Disease

**Table 2:** The correlation between the chemotherapeutic efficacy and Patient type

index		Type number	Benefit number	Benefit percent	*P* (< 0.05)
Patient type	Triploid	16	4	25%	0.048
	Non-triploid	63	54	85.71%	

After first 2-cycles chemotherapy, 21 patients showed progression, and changed treatment strategy. These progressed patients were excluded for the following research. The remaining 58 patients continued second 2-cycles chemotherapy. CTC karyotyping and chemotherapeutic efficacy information after second 2-cycles chemotherapy were shown in Table 3.

**Table 3:** CTC karyotyping and chemotherapeutic efficacy of 58 esophageal cancer patients after second 2-cycles chemotherapy

Sample ID	CTC karyotyping post-chemotherapy	CTC number post-chemotherapy		Non-triploid proportion	chemotherapeutic effect
		Triploid	No-triploid		
2	> 4	0	3	1	SD
3	3	3	0	0	SD
5	3	3	0	0	PR
8	> 4	0	4	1	PD
9	3	2	0	0	PR
11	3, > 4	1	2	0.667	SD
12	3	2	0	0	PR
13	3	2	0	1	SD
14	3, > 4	2	2	0.5	SD
15	3,4	2	3	0.6	SD
16	3	3	0	0	PR
17	3	2	0	0	PR
18	3, 4	5	4	0.444	SD
19	3, > 4	2	1	0.333	PD
20	3	1	0	0	PR
22	3	2	0	0	PR
23	3	3	0	0	PR
26	3,4	2	2	0.5	SD
28	3, 4	1	2	0.667	SD
29	3, > 4	1	2	0.667	PD
30	3	3	0	0	SD
31	4	0	2	1	PD
32	3	1	0	0	PR
34	3	2	0	0	PD
35	4	0	2	1	SD
36	3	1	0	0	PR
38	> 4	0	1	1	SD
39	4	0	1	1	PD
41	3	2	0	0	SD
42	3	1	0	0	PR
44	> 4	0	2	1	SD
45	4	0	2	1	PD
46	3, > 4	1	1	0.5	SD
47	4, > 4	0	10	1	PD
48	3, > 4	1	1	0.5	PR
49	3	2	0	0	PR
51	3, 4	1	3	0.75	PD
52	3	1	0	0	PR
53	> 4	0	1	1	PD
54	3	2	0	0	PR
56	> 4	0	1	1	PD
58	3	1	0	0	PR
59	3,	2	0	0	PR
60	> 4	0	1	1	PD
61	3, 4	2	1	0.333	PR
62	3,4, > 4	2	2	0.5	SD
63	3, > 4	1	1	0.5	SD
65	3, > 4	1	3	0.75	PD
66	3	3	0	0	PD
69	3, 4, > 4	2	4	0.667	SD
70	3, 4, > 4	15	9	0.375	PD
71	3, 4	1	2	0.667	PD
73	3,4, > 4	6	9	0.6	PD
74		0	0	0	PR
75	3	3	0	0	SD
76	3, 4	1	1	0.5	PD
77	3	1	0	0	PR
78	> 4	0	1	1	PD

Note: *PR* Partial Response, *PD* Progressive Disease, *SD* Stable Disease

**Fig. 2:**
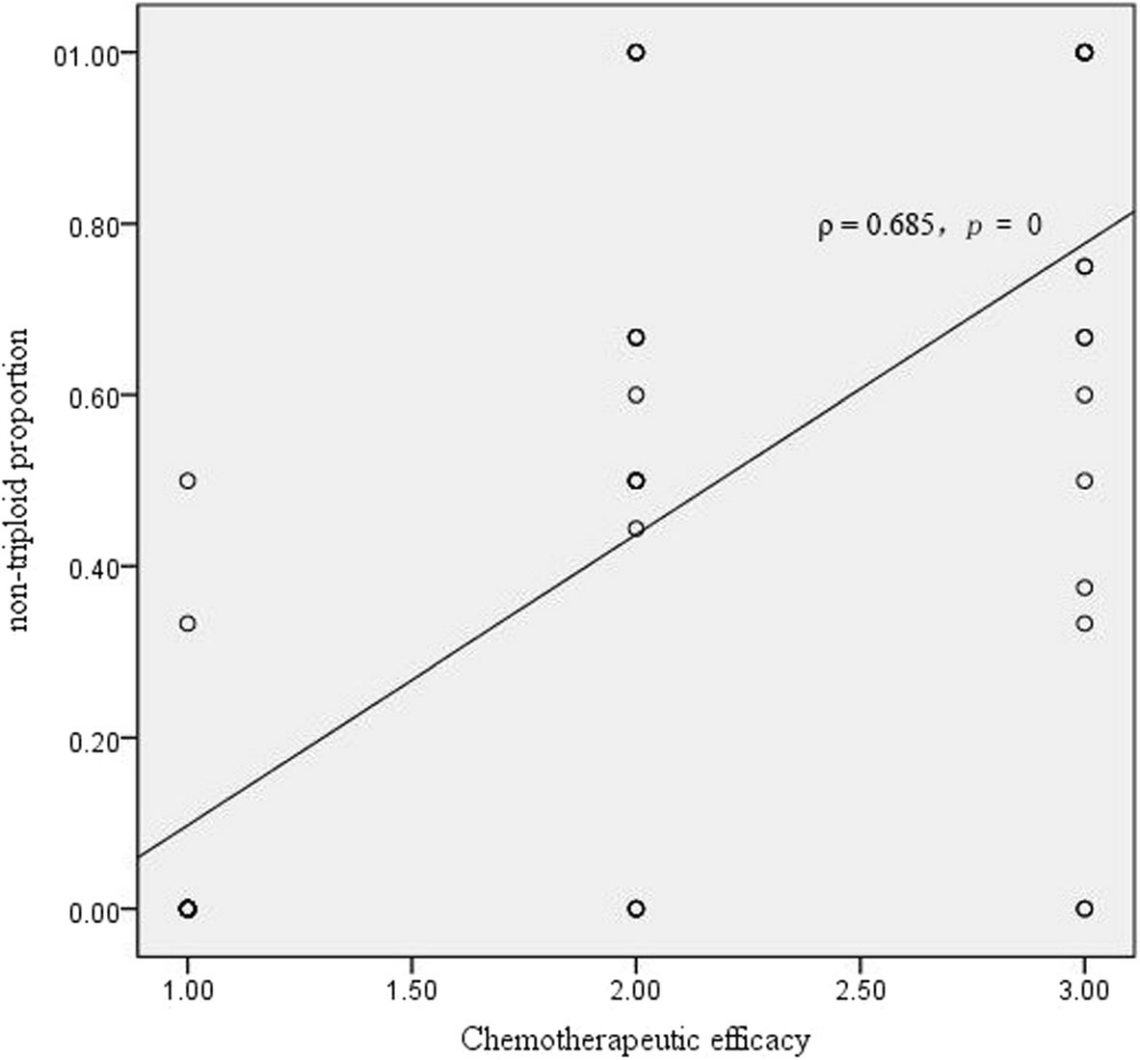
The association between chemotherapeutic efficacy and non-triploid proportion. PR: Partial Response; PD: Progressive Disease; SD: Stable Disease. 1 in X axial represent PR; 2 in X axial represent SD; 3 in X axial represent PD.

The association between chemotherapeutic efficacy and non-triploid proportion showed that chemotherapeutic efficacy was positive correlated with non-triploid proportion (Fig. 2; ρ = 0.685; *p* = 0.000, *P* < 0.05).

## Discussion

In this study, ploidy type of chromosome 8 in CTCs of esophageal cancer patients were detected by SET-iFISH. It was previously confirmed that SET-iFISH was better than conventional detection strategy for measuring CTCs [18, 19].

It has been reported that triploid CTCs displayed characteristic of primary resistance to cisplatin, and tetraploid or more than tetraploid CTCs showed acquired resistance to cisplatin [14]. Lee AJ et al. has been recently demonstrated that drug resistance is likely to be an intrinsic property of aneuploid cancer cells [20]. Therefore, it can conclude that aneuploidy type of the CTC is associated with chemotherapeutic efficacy. In this study, the association between different karyotyping of CTCs measured before or after chemotherapy and chemotherapeutic efficacy were future analysed.

Firstly, the correlation between karyotyping of chromosome 8 in CTCs before chemotherapy and the sensitive of chemotherapy was investigated. Our results also suggested that different aneuploidy in CTCs may play distinct role in drug resistance. Chemotherapeutic efficacy of non-triploid patients displayed significantly higher than triploid patients. So it showed that non-triploid patients had gained more benefits from chemotherapy, triploid patients show resistance to chemotherapy. Therefore, it is concluded that non-triploid subtype could be as a sensitive maker that reflects response to chemotherapy.

The association analysis between chemotherapeutic efficacy and karyotyping of chromosome 8 in CTCs indicated that chemotherapeutic efficacy was positive correlated with non-triploid proportion. When the non-triploid proportion of the patients was higher, the patients are possibly showed PD after multiple chemotherapy. It may be result from the acquired resistance in non-triploid CTC cells after multiple chemotherapy. It is concluded that non-triploid proportion could be used as a maker for chemotherapeutic efficacy. Karyotypic characterization of enriched CTCs during therapy might replace imaging to assess therapeutic resistance, and guide the clinical treatment of cancer patients.

## Conclusion

Molecular mechanisms of aneuploidy of CTCs and its correlation to diverse therapeutic sensitivities need further study. On-going next generation sequencing analysis of a single CTC may reveal genomic landscapes of CTC subtypes [4–24], which should facilitate the elucidation of evolutionary mechanisms involved in therapy resistance.

## Additional file

Additional file 1: **Table S1.** CTC karyotyping and chemotherapeutic efficacy of 79 esophageal cancer patients with first 2-cycles chemotherapy. (DOCX 27 kb)

## Data Availability

The datasets used and analyzed in the current study would be available from the corresponding author upon request.

## Abbreviations

CTCs: circulating tumor cells
SET-iFISH: Subtraction Enrichment-Immunostaining fluorescence in situ hybridizatio
