# Supplementary material for: Karyotyping of circulating tumor cells for predicting chemotherapeutic sensitivity and efficacy in patients with esophageal cancer
Source: BMC Cancer. 2019 Jul 3;19:651. doi: 10.1186/s12885-019-5850-7 (PMC6609398; doi:10.1186/s12885-019-5850-7)
Supplement: Supplementary file 1 — Table S1. CTC karyotyping and chemotherapeutic efficacy of 79 esophageal cancer patients with first 2-cycles chemotherapy. (DOCX 27 kb) [file 12885_2019_5850_MOESM1_ESM.docx]

**Supplementary. Table 1** CTC karyotyping and chemotherapeutic efficacy of 79 esophageal cancer patients with first 2-cycles chemotherapy.

| Sample ID | CTC karyotyping | CTC number pre-chemotherapy | | Triploid proportion | Patient type | Chemotherapeutic efficacy |
| --- | --- | --- | --- | --- | --- | --- |
|  |  | triploid | Non-Triploid |  |  |  |
| 1 | 3, >4 | 5 | 3 | 0.625 | triploid | PD |
| 2 | 3，>4 | 2 | 6 | 0.25 | non-triploid | SD |
| 3 | 3, 4, >4 | 3 | 7 | 0.3 | non-triploid | PR |
| 4 | 0 | 0 | 0 | 0 | non-triploid | PD |
| 5 | 3, 4, >4 | 5 | 15 | 0.25 | non-triploid | PR |
| 6 | 3, | 3 | 0 | 1 | triploid | PD |
| 7 | 3，>4 | 3 | 2 | 0.6 | triploid | PD |
| 8 | 4，>4 | 0 | 4 | 0 | non-triploid | SD |
| 9 | 3, 4 | 5 | 7 | 0.417 | non-triploid | PR |
| 10 | 3，4 | 8 | 4 | 0.667 | triploid | PD |
| 11 | 3, 4，>4 | 2 | 6 | 0.25 | non-triploid | PR |
| 12 | 4，>4 | 0 | 9 | 0 | non-triploid | PR |
| 13 | 3，4 | 3 | 7 | 0.3 | non-triploid | PR |
| 14 | 3，4， | 3 | 3 | 0.5 | non-triploid | SD |
| 15 | 3, 4,>4 | 4 | 6 | 0.4 | non-triploid | SD |
| 16 | 3, 4，>4 | 5 | 8 | 0.417 | non-triploid | PR |
| 17 | 3，>4 | 2 | 2 | 0.5 | non-triploid | PR |
| 18 | 3，>4 | 6 | 5 | 0.545 | non-triploid | SD |
| 19 | 4，>4 | 0 | 6 | 0 | non-triploid | SD |
| 20 | 4，>4 | 0 | 7 | 0 | non-triploid | PR |
| 21 | 3,4 | 3 | 1 | 0.75 | triploid | PD |
| 22 | >4 | 0 | 2 | 0 | non-triploid | PR |
| 23 | 4，>4 | 0 | 6 | 0 | non-triploid | PR |
| 24 | 4， | 0 | 3 | 0 | non-triploid | PD |
| 25 | 3,4，>4 | 32 | 20 | 0.615 | triploid | PD |
| 26 | 4，>4 | 0 | 2 | 0 | non-triploid | SD |
| 27 | 3，>4 | 4 | 2 | 0.667 | triploid | PD |
| 28 | 4 | 0 | 1 | 0 | non-triploid | PR |
| 29 | >4 | 0 | 2 | 0 | non-triploid | PR |
| 30 | 3，4 | 3 | 3 | 0.5 | non-triploid | SD |
| 31 | 4，>4 | 0 | 4 | 0 | non-triploid | SD |
| 32 | >4 | 0 | 3 | 0 | non-triploid | PR |
| 33 | 3，>4 | 2 | 1 | 0.667 | triploid | PD |
| 34 | 4，>4 | 0 | 5 | 0 | non-triploid | SD |
| 35 | >4 | 0 | 1 | 0 | non-triploid | SD |
| 36 | >4 | 0 | 3 | 0 | non-triploid | PR |
| 37 | 3,4，>4 | 20 | 12 | 0.625 | triploid | PD |
| 38 | 0 | 0 | 0 | 0 | non-triploid | PR |
| 39 | 4，>4 | 0 | 4 | 0 | non-triploid | SD |
| 40 | 3，>4 | 3 | 1 | 0.75 | triploid | PD |
| 41 | 3，>4 | 3 | 1 | 0.75 | triploid | SD |
| 42 | 3，4 | 1 | 3 | 0.25 | non-triploid | PR |
| 43 | 3，>4 | 2 | 6 | 0.25 | non-triploid | PD |
| 44 | 4，>4 | 0 | 6 | 0 | non-triploid | SD |
| 45 | 3，4 | 2 | 2 | 0.5 | non-triploid | SD |
| 46 | >4 | 0 | 2 | 0 | non-triploid | SD |
| 47 | 3，>4 | 3 | 3 | 0.5 | non-triploid | SD |
| 48 | 3，4 | 2 | 3 | 0.4 | non-triploid | PR |
| 49 | 4，>4 | 0 | 5 | 0 | non-triploid | PR |
| 50 | 3 | 3 | 0 | 1 | triploid | PD |
| 51 | 3,4 | 2 | 3 | 0.4 | non-triploid | PR |
| 52 | >4 | 0 | 2 | 0 | non-triploid | PR |
| 53 | 4 | 0 | 3 | 0 | non-triploid | PR |
| 54 | 3，>4 | 3 | 4 | 0.429 | non-triploid | PR |
| 55 | 3，>4 | 5 | 5 | 0.5 | non-triploid | PD |
| 56 | 4 | 0 | 2 | 0 | non-triploid | SD |
| 57 | 3,4，>4 | 4 | 5 | 0.444 | non-triploid | PD |
| 58 | 3 | 1 | 0 | 1 | triploid | SD |
| 59 | 3，>4 | 2 | 1 | 0.667 | triploid | SD |
| 60 | 4，>4 | 0 | 3 | 0 | non-triploid | PR |
| 61 | 4，>4 | 0 | 6 | 0 | non-triploid | PR |
| 62 | 4 | 0 | 4 | 0 | non-triploid | PR |
| 63 | 4 | 0 | 2 | 0 | non-triploid | PR |
| 64 | 4 | 0 | 6 | 0 | non-triploid | PD |
| 65 | 4，>4 | 0 | 5 | 0 | non-triploid | SD |
| 66 | 3，4 | 2 | 1 | 0.667 | triploid | SD |
| 67 | >4 | 0 | 3 | 0 | non-triploid | PD |
| 68 | 3 | 2 | 0 | 1 | triploid | PD |
| 69 | >4 | 0 | 3 | 0 | non-triploid | SD |
| 70 | 4 | 0 | 2 | 0 | non-triploid | SD |
| 71 | 3,4 | 3 | 3 | 0.5 | non-triploid | SD |
| 72 | 4，>4 | 0 | 4 | 0 | non-triploid | PD |
| 73 | 3，>4 | 1 | 3 | 0.25 | non-triploid | PR |
| 74 | 4，>4 | 0 | 2 | 0 | non-triploid | PR |
| 75 | 3，4 | 1 | 2 | 0.333 | non-triploid | SD |
| 76 | 3，>4 | 1 | 2 | 0.333 | non-triploid | SD |
| 77 | 4 | 0 | 2 | 0 | non-triploid | PR |
| 78 | 4，>4 | 0 | 2 | 0 | non-triploid | PR |
| 79 | 3，4，>4 | 9 | 11 | 0.45 | non-triploid | PD |
